# Higher dietary fibre intake is associated with increased skeletal muscle mass and strength in adults aged 40 years and older

**DOI:** 10.1002/jcsm.12820

**Published:** 2021-09-29

**Authors:** James Frampton, Kevin G. Murphy, Gary Frost, Edward S. Chambers

**Affiliations:** ^1^ Section for Nutrition Research, Department of Metabolism, Digestion and Reproduction, Faculty of Medicine Imperial College London London UK; ^2^ Section of Endocrinology and Investigative Medicine, Department of Metabolism, Digestion and Reproduction, Faculty of Medicine Imperial College London London UK

**Keywords:** Fibre, Diet, Skeletal muscle, NHANES

## Abstract

**Background:**

Skeletal muscle mass begins to decline from 40 years of age. Limited data suggest that dietary fibre may modify lean body mass (BM), of which skeletal muscle is the largest and most malleable component. We investigated the relationship between dietary fibre intake, skeletal muscle mass and associated metabolic and functional parameters in adults aged 40 years and older.

**Methods:**

We analysed cross‐sectional data from the US National Health and Nutrition Examination Survey between 2011 and 2018 from adults aged 40 years and older. Covariate‐adjusted multiple linear regression analyses were used to evaluate the association between dietary fibre intake and BM components (BM, body mass index [BMI], total lean mass, appendicular lean mass, bone mineral content, total fat, trunk fat; *n* = 6454), glucose homeostasis (fasting glucose, fasting insulin, HOMA2‐IR; *n* = 5032) and skeletal muscle strength (combined grip strength; *n* = 5326). BM components and skeletal muscle strength were expressed relative to BM (per kg of BM).

**Results:**

Higher intakes of dietary fibre were significantly associated with increased relative total lean mass (β: 0.69 g/kg BM; 95% CI, 0.48–0.89 g/kg BM; *P* < 0.001), relative appendicular lean mass (β: 0.34 g/kg BM; 95% CI, 0.23–0.45 g/kg BM; *P* < 0.001), relative bone mineral content (β: 0.05 g/kg BM; 95% CI, 0.02–0.07 g/kg BM; *P* < 0.001) and relative combined grip strength (β: 0.002 kg/kg BM; 95% CI, 0.001–0.003 kg/kg BM; *P* < 0.001).

Conversely, higher dietary fibre intakes were significantly associated with a lower BM (β: −0.20; 95% CI, −0.28 to −0.11 kg; *P* < 0.001), BMI (β: −0.08 kg/m^2^; 95%CI, −0.10 to −0.05 kg/m^2^), relative total fat (β: −0.68 g/kg BM; 95% CI, −0.89 to −0.47 g/kg BM; *P* < 0.001), relative trunk fat (β: −0.48 g/kg BM; 95%CI, −0.63 to −0.33 g/kg; *P* < 0.001), fasting glucose (β: −0.01 mmol/L; 95% CI, −0.02 to −0.00 mmol/L; *P* = 0.017), fasting insulin (β: −0.71 pmol/L; 95% CI, −1.01 to −0.41 pmol/L; *P* < 0.001) and HOMA2‐IR (β: −0.02 AU; 95% CI, −0.02 to −0.01 AU; *P* < 0.001).

**Conclusions:**

Higher dietary fibre intakes are associated with a lower BM and enhanced body composition, characterized by a reduction in fat mass and an increase in lean mass. Higher dietary fibre intakes were also associated with improvements in glucose homeostasis and skeletal muscle strength. Increasing dietary fibre intake may be a viable strategy to prevent age‐associated declines in skeletal muscle mass.

## Introduction

Increased dietary fibre intake is associated with a reduction in cardiometabolic disease risk and all‐cause mortality.^1^ This relationship is likely mediated in part by the effect of dietary fibre on decreasing body mass.^2^ However, human intervention studies demonstrate that increasing dietary fibre intake can lower adiposity without changing body mass,^3^, ^4^ indicating that increased dietary fibre intake can also raise lean body mass. The improvements in cardiometabolic outcomes in these studies are typically attributed to a decrease in total and regional fat mass, although the impact of raised lean body mass is often neglected.

Skeletal muscle is the largest and most malleable component of lean body mass (accounting for ~50% of lean body mass^5^), and hence, changes in lean body mass can be primarily attributed to skeletal muscle mass. The maintenance of skeletal muscle is fundamental to locomotion, energy homeostasis and overall quality of life.^6^, ^7^ Skeletal muscle is of particular importance to cardiometabolic disease risk, acting as the primary site of insulin‐stimulated glucose uptake in the human body.^8^ Indeed, skeletal muscle insulin resistance has been proposed as the principal defect in Type 2 diabetes.^9^ Skeletal muscle mass and associated strength, however, begin to decline after the fifth decade of life,^10^, ^11^ with adults losing ~20% of their skeletal muscle mass between the ages of 40 and 80 years^12^; this directly contributes to the metabolic dysregulation and functional impairments observed in the elderly population. Consequently, strategies that promote or protect skeletal muscle mass in middle age are needed to help maintain functional independence and cardiometabolic health in later life.

In most countries, there is a substantial ‘dietary fibre gap’ between reported intakes in adult populations and the amount recommended by national dietary guidelines and health institutes. For example, in the United States, mean intake is approximately 17 g/day, and the recommended intake of 14 g/1000 kcal (∼28–34 g/day) is met by <10% adults.^13^, ^14^ However, the relationship between dietary fibre intake and skeletal muscle mass, strength and associated glycaemic parameters in adults at increased risk of skeletal muscle atrophy is currently unknown.

We therefore used nationally representative US population data from 2011 to 2018 to investigate associations between dietary fibre intake and body mass components, glucose homeostasis and skeletal muscle strength in adults aged 40 years and older. We hypothesized that a higher intake of dietary fibre would be associated with improved body composition (increased lean body mass *and* decreased fat mass). Furthermore, we hypothesized that this improvement in body composition would be paralleled by an enhancement in glucose homeostasis and skeletal muscle strength with increasing dietary fibre intake. The findings from this study will help to inform whether interventions to raise dietary fibre intake are a worthwhile avenue for the prevention of age‐associated declines in skeletal muscle mass and strength.

## Methods

This manuscript was written in accordance with the Strengthening the Reporting of Observational Studies in Epidemiology (STROBE) statement for cross‐sectional studies.^15^

### Study design

This study used publicly available data from the National Health and Nutrition Examination Survey (NHANES). NHANES is a continual cross‐sectional survey designed to evaluate the health and nutritional status of the civilian, non‐institutionalized population of the United States. NHANES employs a complex, multistage, probability sampling design with oversampling of specified population subgroups to increase the reliability and precision of estimates. Participants completed in‐home interviews, physical examinations (including the collection of blood and urine samples) and dietary interviews. Comprehensive descriptions of methodology and data collection are provided elsewhere.^16^ NHANES was conducted in line with the Declaration of Helsinki and approved by the National Center for Health Statistics Ethics Review Board. Informed consent was obtained from all participants prior to involvement.

### Study participants

This study involved participants aged ≥40 years from four consecutive survey cycles: 2011–2012, 2013–2014, 2015–2016 and 2017–2018 (each survey cycle represents an independent sample from the population). A cut‐off age of ≥40 years old was chosen based on evidence that loss of skeletal muscle mass may begin to accelerate after this age.^10^, ^11^

### Dietary intake

NHANES uses in‐person 24‐h dietary recall interviews using the US Department of Agriculture (USDA) Automated Multiple‐Pass Method to quantify food and beverage intake. Nutrient intakes are then calculated from food and beverage data using the USDA Food and Nutrient Database for Dietary Studies.^17^, ^18^ Energy intake (kcal), dietary fibre, alcohol and macronutrient intake (grams) per day were then determined. The accuracy of this method has been repeatedly assessed and shown to produce estimates within 10% of true intake.^19^, ^20^

### Outcomes

Outcomes included in this study were body mass, body mass index (BMI), total lean mass (excluding bone mineral content), appendicular lean mass (excluding bone mineral content), bone mineral content, total fat, trunk fat, fasting glucose, fasting insulin, insulin resistance as calculated by the updated homeostasis model assessment (HOMA2‐IR) and combined grip strength. However, several outcomes were only measured in specific survey cycles and subsamples. Analyses were therefore performed using three distinct datasets.

#### Body mass components dataset

Body mass (kg) and BMI (kg/m^2^) were measured for all survey cycles (2011–2018) and collected in the mobile examination centre.

For all survey cycles (2011–2018), total lean mass (g), bone mineral content (g), total fat (g) and trunk fat (g) were measured using in eligible participants. Appendicular lean mass (g), a well‐recognized proxy for skeletal muscle mass,^21^ was calculated by summing the lean mass (excluding bone mineral content) of the right and left leg and the right and left arm as measured by dual‐energy x‐ray absorptiometry (DEXA). Bone mineral content was also included due to the well‐documented coupling of bone and skeletal muscle wasting during the ageing process.^22^ All variables measured by DEXA were expressed relative to body mass (g per kg of body mass; g/kg BM) to account for the influence of body mass on differences in these outcomes. Participants older than 59 years were not eligible for DEXA measurements, and therefore, the age range of all body mass components dataset variables (including body mass and BMI) was limited to 40–59 years.

#### Glucose homeostasis dataset

For all survey cycles (2011–2018), fasting measures of glucose (mmol/L) and insulin (pmol/L) were taken in a subsample of eligible participants. Fasting glucose and insulin concentrations were then used to calculate HOMA2‐IR^23^ (arbitrary units; AU).

#### Skeletal muscle strength dataset

For survey cycles 2011–2012 and 2013–2014 only, skeletal muscle strength was measured through a grip test using a handgrip dynamometer in eligible participants. Grip strength is a widely used objective measure of global skeletal muscle strength that predicts functional impairment and all‐cause mortality.^24^ Combined grip strength (kg) was calculated by summing the largest reading from the right and left hand and expressed relative to body mass (kg/kg BM).

### Covariates

Covariates included in this study were sex, age, ethnicity, social economic status, smoking status, sedentary activity, total daily energy intake, total alcohol intake and the percentage of energy contributed by fat, carbohydrate and protein to total daily energy intake. All covariates were assumed to confound the relationship between dietary fibre intake and outcome variables.

Age (years), sex (male, female), ethnicity, socio‐economic status and smoking status (smoker, non‐smoker) were self‐reported during in‐home interviews. Ethnic groups included Mexican American, other Hispanic, non‐Hispanic White, non‐Hispanic Black and other. Social economic status was classified using the ratio of family income to poverty (PIR), with participants being categorized as low (PIR ⩽ 1.3), middle (PIR > 1.3 to ⩽3.5) or high (PIR > 3.5) socio‐economic status. Sedentary activity (minutes) was calculated from the physical activity questionnaire and was preferred over other measures of physical activity due to its high response rate. Total daily energy intake (kcal) and total daily alcohol intake (grams) were derived from the 24‐h dietary recall (first day). The percentage of energy contributed by fat (%), carbohydrate (%) and protein (%) to total daily energy intake was calculated using the amount of each macronutrient consumed (g) derived from the 24‐h dietary recall (first day). This amount was multiplied by its energy content (4 kcal/g for carbohydrate and protein, 9 kcal/g for fat) and then divided by total daily energy intake to provide a percentage.

### Statistical analysis

All statistical procedures were performed in Stata 16 (StataCorp, USA) and accounted for the complex survey design used in the NHANES (stratification and clustering). Taylor series linearization methods were used for variance estimation.

Four‐ (2011–2014) and 8‐year sample weights (2011–2018) were generated by combining 2‐year sample weights for each survey cycle as previously described.^25^ Sample weights were applied to all analyses to produce nationally representative estimates. Eight‐year dietary Day 1 sample weights were used for the body mass components dataset, 8‐year fasting subsample weights were used for the glucose homeostasis dataset, and 4‐year dietary Day 1 sample weights were used for the skeletal muscle strength dataset.

#### Multiple imputation of missing data

As recommended in the NHANES analytic guidelines,^25^ outcome variables for which >10% of data are missing for eligible participants require adjustment prior to analysis. Consequently, multiple imputation with chained equations was used to impute missing values for total lean mass, right leg lean mass, left leg lean mass, right arm lean mass, left arm lean mass, total fat and trunk fat. Full details of the imputation model are provided in *Appendix* *S1*.

#### Participant eligibility

For the body mass components dataset, participants who were not eligible for DEXA measurements or did not undertake the 24‐h dietary recall interviews (Day 1) were excluded from analysis. Participants with missing values for body mass components outcomes (body mass, BMI, trunk fat, total fat, total lean mass, appendicular lean mass, bone mineral content), dietary fibre intake and/or covariates were, however, not excluded, as multiply imputed values were used. This resulted in 6454 eligible participants for the body mass components dataset (*Appendix* *S2*).

Within the glucose homeostasis dataset, participants with missing data for fasting glucose, fasting insulin, HOMA2‐IR, dietary fibre intake or covariates were excluded from analysis. Similarly, participants with missing data for combined grip strength, dietary fibre intake or covariates were excluded from analysis within the skeletal muscle strength dataset. This resulted in 5032 and 5326 eligible participants for the glucose homeostasis and skeletal muscle strength datasets, respectively (*Appendix* *S2*).

#### Association of dietary fibre intake with outcomes

Simple and multiple linear regression analyses were used to examine the association between dietary fibre intake (treated as continuous variable) and all outcome variables. Model 1 was an unadjusted model. Model 2 adjusted for socio‐demographic and behavioural variables: gender, age, ethnicity, socio‐economic status, smoking status and sedentary activity. Model 3 adjusted for socio‐demographic, behavioural and dietary variables: gender, age, ethnicity, socio‐economic status, smoking status, sedentary activity, total energy intake, total alcohol intake, percent energy from protein, percent energy from carbohydrate and percent energy from fat. The results from Model 3 are presented as the main results. For body mass components and skeletal muscle strength outcomes that showed a significant association with dietary fibre intake in Model 3, a further model was created (Model 4). Model 4 was identical to Model 3, except that it was adjusted for an additional covariate (HOMA2‐IR) to evaluate the role of insulin resistance in the relationship between dietary fibre and outcomes in the body mass components and skeletal muscle strength datasets. Restricted cubic splines were used to model non‐linear relationships between dietary fibre intake and outcomes, with three knots placed at the 10th, 50th and 90th percentiles.^26^

Results from regression analyses are presented as regression coefficients (β) and corresponding 95% confidence intervals (CI), *P*‐values and coefficients of determination (*R* ^2^). Significant differences were defined as *P* < 0.05.

## Results

Population‐weighted means of socio‐demographic and behavioural characteristics for each dataset are presented in *Table* *1*. Mean dietary fibre intake was ≈17 g/day for all datasets.

**Table 1:** Population‐weighted socio‐demographic and behavioural characteristics of the glucose homeostasis, body mass components and skeletal muscle function datasets

Characteristics	Body mass components dataset^a^	Glucose homeostasis dataset^b^	Skeletal muscle strength dataset^c^
	*n* = 6454	*n* = 5032	*n* = 5326
Sex (%)			
Male	48.3 (0.9)	47.9 (0.6)	48.0 (0.9)
Female	51.7 (0.9)	52.1 (0.6)	52.0 (0.9)
Age (years)	49.9 (0.1)	58.0 (0.3)	57.7 (0.2)
	[40–59]	[40–80^d^]	[40–80^d^]
Ethnicity (%)			
Mexican American	8.5 (1.0)	6.1 (0.7)	5.5 (1.0)
Other Hispanic	6.1 (0.7)	5.0 (0.6)	4.5 (0.7)
Non‐Hispanic White	64.3 (1.9)	72.0 (1.7)	73.1 (2.3)
Non‐Hispanic Black	11.8 (1.0)	9.7 (0.9)	10.3 (1.4)
Other	9.4 (0.6)	7.3 (0.5)	6.6 (0.6)
Socio‐economic status (%)			
Low	20.2 (1.3)	18.3 (1.2)	19.5 (1.8)
Middle	32.7 (1.3)	35.6 (1.2)	34.1 (1.4)
High	47.1 (1.8)	46.1 (1.8)	46.4 (2.4)
Sedentary activity (min/day)	389.2 (5.0)	391.2 (4.5)	407.3 (4.9)
Total daily energy intake (kcal)	2180 (18)	2126 (16)	2077 (18)
Energy contribution from carbohydrate (%)	47.3 (0.3)	47.4 (0.3)	48.1 (0.3)
Energy contribution from fat (%)	34.7 (0.2)	35.2 (0.2)	34.0 (0.2)
Energy contribution from protein (%)	15.8 (0.1)	15.8 (0.1)	15.9 (0.1)
Fibre (g)	17.4 (0.2)	17.2 (0.2)	17.6 (0.3)

Data are %N (SE) or mean (SE) [range].

^a^ Eight‐year dietary Day 1 sample weights (2011–2018).

^b^ Eight‐year fasting subsample weights (2011–2018).

^c^ Four‐year dietary Day 1 sample (2011–2014).

^d^ Individuals aged over 80 years were top‐coded as 80 years.

### Dietary fibre intake and body mass components

Dietary fibre intake showed a significant negative association with body mass and BMI (*Table* *2*). Assuming linearity, each 5 g increase in daily dietary fibre intake was associated with a decrease of 0.98 kg (95% CI, −1.39 to −0.55 kg) and 0.38 kg/m^2^ (95% CI, −0.52 to −0.24 kg/m^2^) in body mass and BMI, respectively.

**Table 2:** Simple and multiple linear regression analyses of dietary fibre intake (g/day) and all outcomes

Outcomes	Model 1^a^			Model 2^b^			Model 3^c^		
	β (95% CI)	*P*‐value	*R* ^2^	β (95% CI)	*P*‐value	R^2^	β (95% CI)	*P*‐value	*R* ^2^
Body mass (kg)	0.05 (−0.01, 0.11)	0.093	<0.01	−0.04 (−0.10, 0.01)	0.127	0.16	−0.20 (−0.28, −0.11)	<0.001	0.18
BMI (kg/m^2^)	−0.03 (−0.05, −0.01)	0.002	<0.01	−0.03 (−0.05, −0.01)	0.002	0.07	−0.08 (−0.10, −0.05)	<0.001	0.09
Relative total lean mass (g/kg BM)	1.46 (1.20, 1.71)	<0.001	0.04	0.59 (0.44, 0.73)	<0.001	0.56	0.69 (0.48, 0.89)	<0.001	0.57
Relative appendicular lean mass (g/kg BM)	0.84 (0.70, 0.97)	<0.001	0.04	0.33 (0.25, 0.40)	<0.001	0.62	0.34 (0.23, 0.45)	<0.001	0.63
Relative bone mineral content (g/kg BM)	0.04 (0.02, 0.06)	<0.001	<0.01	0.02 (0.01, 0.04)	0.013	0.10	0.05 (0.02, 0.07)	<0.001	0.11
Relative total fat (g/kg BM)	−1.46 (−1.73, −1.19)	<0.001	0.04	−0.59 (−0.74, −0.44)	<0.001	0.55	−0.68 (−0.89, −0.47)	<0.001	0.56
Relative trunk fat (g/kg BM)	−0.67 (−0.80, −0.55)	<0.001	0.03	−0.44 (−0.54, −0.33)	<0.001	0.28	−0.48 (−0.63, −0.33)	<0.001	0.29
Fasting glucose (mmol/L)	−0.00 (−0.01, 0.01)	0.934	<0.01	−0.00 (−0.01, 0.00)	0.143	0.03	−0.01 (−0.02, −0.00)	0.017	0.04
Fasting insulin (pmol/L)	0.02 (−0.20, 0.25)	0.847	<0.01	−0.14 (−0.37, 0.09)	0.249	0.03	−0.71 (−1.01, −0.41)	<0.001	0.05
HOMA2‐IR (AU)	0.00 (−0.00, 0.01)	0.862	<0.01	−0.00 (−0.01, 0.00)	0.228	0.03	−0.02 (−0.02, −0.01)	<0.001	0.05
Relative combined grip strength (kg/kg BM)	0.004 (0.003, 0.005)	<0.001	0.03	0.002 (0.001, 0.003)	<0.001	0.39	0.002 (0.001, 0.003)	<0.001	0.40

AU, arbitrary units; BMI, body mass index; HOMA2‐IR, updated homeostasis model assessment—insulin resistance.

^a^ Unadjusted model.

^b^ Adjusted for gender, age, ethnicity, socio‐economic status, smoking status and sedentary activity.

^c^ Adjusted for gender, age, ethnicity, socio‐economic status, smoking status, sedentary activity, total energy intake, total alcohol intake, percent energy from protein, percent energy from carbohydrate and percent energy from fat.

Dietary fibre intake showed a significant positive association with relative total lean mass, relative appendicular lean mass and relative bone mineral content (*Table* *2*). Assuming linearity, each 5 g increase in daily dietary fibre intake was associated with an increase of 3.44 g/kg BM (95% CI, 2.40–4.47 g/kg BM), 1.69 g/kg BM (95% CI, 1.13–2.25 g/kg BM) and 0.23 g/kg BM (95% CI, 0.09 to 0.36 g/kg BM) in relative total lean mass, relative appendicular lean mass and relative bone mineral content, respectively.

Dietary fibre intake showed a significant negative association with relative total fat and relative trunk fat (*Table* *2*). Assuming linearity, each 5 g increase in daily dietary fibre intake was associated with a decrease of 3.40 g/kg BM (95% CI, −4.45 to −2.36 g/kg BM) and 2.40 g/kg BM (95% CI, −3.14 to −1.67 g/kg BM) in relative total fat and relative trunk fat, respectively.

Including HOMA2‐IR as an additional covariate (Model 4) produced comparable findings to the main results (Model 3) for all outcomes in the body mass components dataset; direction, magnitude and statistical significance of regression coefficients were largely unaltered (*Appendix* *S3*).

### Dietary fibre intake and glucose homeostasis

Dietary fibre intake showed a significant negative association with fasting glucose, fasting insulin and HOMA2‐IR (*Table* *2*). Assuming linearity, each 5 g increase in dietary fibre intake was associated with a decrease of 0.04 mmol/L (95% CI, −0.08 to −0.01 mmol/L), 3.55 pmol/L (95% CI, −5.03 to −2.07 pmol/L) and 0.08 AU (95% CI, −0.12 to −0.05 AU) in fasting glucose, fasting insulin and HOMA2‐IR, respectively.

### Dietary fibre intake and skeletal muscle strength

Dietary fibre intake showed a significant positive association with relative combined grip strength (*Table* *2*). Assuming linearity, each 5 g increase in daily dietary fibre intake was associated with an increase of 0.012 kg/kg BM (95% CI, 0.006–0.018 kg/kg BM) in relative combined grip strength.

Including HOMA2‐IR as an additional covariate (Model 4) produced comparable findings to the main results (Model 3) for relative combined grip strength; direction, magnitude and statistical significance of the regression coefficient was largely unaltered (*Appendix* *S3*).

### Dose–response relationships

**Figure 1:**
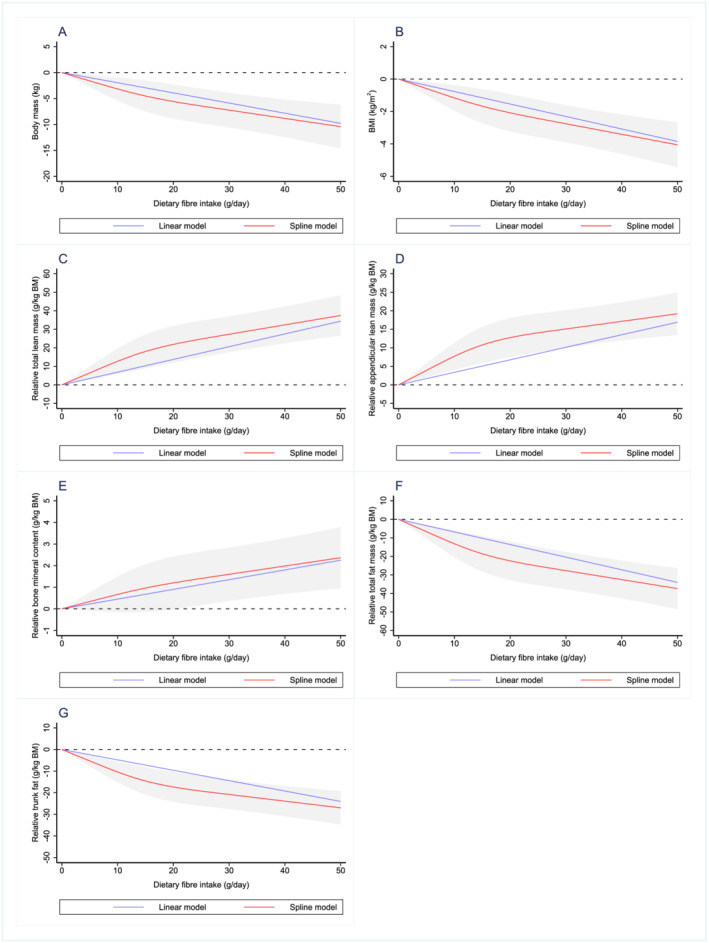
Dose–response relationship between dietary fibre intake and (A) body mass, (B) BMI, (C) relative total lean mass, (D) relative appendicular lean mass, (E) relative bone mineral content, (F) relative total fat and (G) relative trunk fat. Values represent difference in predicted response in reference to a dietary fibre intake of zero. Red and blue solid lines represent linear and restricted cubic spline models, respectively. Black dotted line indicates no change from a dietary fibre intake of zero. Linear and spline models were adjusted for gender, age, ethnicity, socio‐economic status, smoking status, sedentary activity, total energy intake, total alcohol intake, percent energy from protein, percent energy from carbohydrate and percent energy from fat. Grey‐shaded area represents 95% confidence interval from restricted cubic spline model predictions.

**Figure 2:**
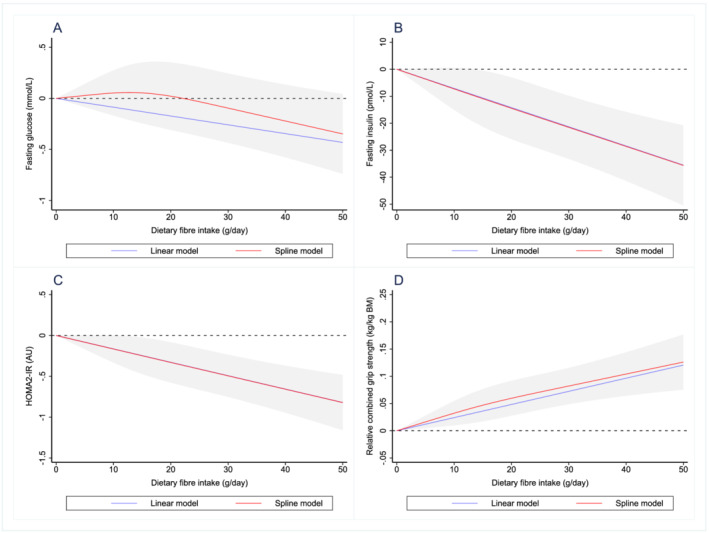
Dose–response relationship between dietary fibre intake and (A) fasting glucose, (B) fasting insulin, (C) HOMA2‐IR and (D) relative combined grip strength. Values represent difference in predicted response in reference to a dietary fibre intake of zero. Red and blue solid lines represent linear and restricted cubic spline models, respectively. Black dotted line indicates no change from a dietary fibre intake of zero. Linear and spline models were adjusted for gender, age, ethnicity, socio‐economic status, smoking status, sedentary activity, total energy intake, total alcohol intake, percent energy from protein, percent energy from carbohydrate and percent energy from fat. Grey‐shaded area represents 95% confidence interval from restricted cubic spline model predictions.

Dose–response relationships between dietary fibre intake and all outcomes are shown in *Figures* *1* and *2*. Outcomes were also analysed across quarters of daily dietary fibre intake and level of dietary guideline adherence (meeting recommended dietary fibre intake vs. not meeting recommended dietary fibre intake), producing comparable findings to the main results (*Appendices* *S4* and *S5*).

## Discussion

The aim of the present analysis was to examine the relationship between dietary fibre intake and body mass components, glucose homeostasis and skeletal muscle strength in adults aged 40 years and older. Using nationally representative data of the US population from the NHANES, we show that higher dietary fibre intakes in adults at increased risk of skeletal muscle mass loss are associated with an increase in relative total lean mass, relative appendicular lean mass, relative bone mineral content and relative combined grip strength. We also demonstrate that higher dietary fibre intakes are associated with a lower body mass, BMI, fasting glucose, fasting insulin, HOMA2‐IR, relative fat mass and relative trunk fat mass. To our knowledge, the association between dietary fibre and skeletal muscle mass and function has not been previously investigated in a cohort of this size and scope. Previous research in this area has used relatively small sample sizes and a narrower age range of study participants (65–79 years) and did not adjust for important covariates (e.g. socio‐economic status, physical activity level, smoking status and alcohol intake).^27^ The findings of the present study indicate that the divergent changes in the lean and fat components of body mass associated with higher dietary fibre intakes are also allied to improvements in glucose homeostasis and skeletal muscle strength. Consequently, interventions that aim to increase dietary fibre intake, via supplementation or increased consumption of high‐fibre foods, may be a viable strategy to prevent age‐associated declines in lean body mass and associated strength.

The finding that a higher dietary fibre intake is associated with a lower body mass and BMI is consistent with prior observational, prospective and interventional studies.^1^ Assuming a linear relationship between dietary fibre intake and body mass, our analysis suggests that the average US citizen (dietary fibre intake of ∼15 g/day) would be ∼2 to ∼4 kg heavier than a similar individual (matched by covariates included in Model 3) who met the recommended dietary fibre intake (∼30 g/day). The effect of dietary fibre on fat mass observed in the present analysis is also supported by previous research investigating this relationship.^28^ Furthermore, increased total fat and trunk fat are associated with insulin resistance,^29^ and therefore, lower total fat and trunk fat likely contribute to the relationship between higher dietary fibre intakes and lower fasting glucose, fasting insulin and HOMA2‐IR.

Alongside its favourable effects on body mass and fat mass, higher dietary fibre intakes are associated with an increase in relative total lean mass and relative appendicular lean mass. This is congruent with the limited available evidence in humans showing fibre supplementation can modify lean body mass.^3^, ^4^, ^30^ Indeed, diets that have shown promise in preventing the age‐related decline in skeletal muscle mass are typically characterized by a high intake of fibre‐rich foods such as fruits, vegetables and wholegrains.^31^ The increase in relative appendicular lean mass (a proxy for skeletal muscle mass) with increasing dietary fibre intake likely contributes to the positive relationship between dietary fibre intake and improvements in glucose homeostasis outcomes (alongside the concurrent reduction in relative fat mass) as skeletal muscle is the primary site of glucose storage.^32^ However, due to the scarcity of research in this area, the mechanisms responsible for the putative relationship between dietary fibre and skeletal muscle mass have been little explored or discussed. As dietary fibre exhibits pleiotropic effects on the human body, it may influence skeletal muscle mass via multiple avenues. For example, dietary fibre supplementation has been shown to decrease insulin resistance^33^, ^34^ (in agreement with the findings of the present analysis) and concentrations of pro‐inflammatory cytokines^35^, ^36^—two factors that increase muscle protein loss^37^, ^38^ and are implicated in the age‐related decline in skeletal muscle mass and function.^39^ Nevertheless, our results show that after adjusting for HOMA2‐IR, the relationship between dietary fibre, body composition and skeletal muscle strength is largely unchanged. This suggests that any potential effect of dietary fibre on these outcomes is not entirely dependent on improvements in insulin resistance and fibre‐induced changes in body composition and skeletal muscle strength may be driven via alternate mechanisms. Dietary fibre intake has a major impact on the composition and metabolic activity of the gut microbiome. For example, higher dietary fibre intake raises the abundance of saccharolytic gut bacteria and production of short‐chain fatty acids (SCFAs),^40^, ^41^ which have recently been proposed as regulators of skeletal muscle mass, metabolism and function.^42^. SCFAs are also implicated in energy balance and glucose homeostasis^43^ and may therefore contribute to the relationship between higher dietary fibre intakes and lower body mass, BMI, fasting glucose, fasting insulin and HOMA2‐IR.

The positive association between dietary fibre intake and relative combined grip strength is likely explained by the positive association between dietary fibre intake and relative appendicular lean mass, considering the strong linear relationship between skeletal muscle mass and strength.^44^ This is in line with previous work showing that elderly individuals in the highest tertile of skeletal muscle mass had significantly higher relative handgrip strength and physical function test scores than individuals in the lowest tertile^27^ and that chronic fibre supplementation (comprising inulin and fructooligosaccharides) could significantly improve hand grip strength in this same population.^45^ Collectively, this would suggest that dietary strategies that impact skeletal muscle mass likely have downstream effects on skeletal muscle strength and function and may therefore have important implications for the maintenance of skeletal muscle mass and function during the ageing process.

Dose–response curves suggest that most outcomes display an approximate linear relationship with dietary fibre intake and display no sign of a plateau over the range of dietary fibre intake explored. Despite some outcomes in the body mass components datasets showing a possible decrease in the strength of the relationship when dietary fibre intakes exceed 20 g/day, the data presented in the current analysis suggest that increasing dietary fibre intake above the current population intake (and towards recommended levels^46^) would likely be associated with improvements in body composition, glucose homeostasis and strength in adults aged 40 years and older.

Nevertheless, the present analysis has several limitations. Firstly, this analysis only includes non‐institutionalized participants from the United States, and therefore, it is unknown whether the relationship between dietary fibre and skeletal muscle mass and function are present in other populations or countries. The findings from this analysis were also partially limited by the age restrictions imposed on the DEXA measurements, resulting in total lean mass, appendicular lean mass, total fat and trunk fat only being measured in individuals aged up to 59 years old. This may be problematic if the effect of dietary fibre on body composition is mediated by the gut microbiota, as reports have consistently identified age‐related alterations to the composition and metabolic activity of the faecal microbiota.^47^, ^48^ However, previous research has shown that dietary fibre intake is a major factor influencing the composition of the gut microbiota in the elderly and correlates with decreased incidence of frailty.^49^ Furthermore, the response of the gut microbiota to dietary fibre ingestion was shown to be comparable between middle‐age (30–50 years) and older (≥70 years) adults.^50^ Thus, any association between dietary fibre and body composition produced by the gut microbiota is still likely to be present in adults aged >60 years. A further limitation of this analysis was the approach employed for dietary assessment. Dietary fibre intake and other diet‐related variables were calculated using 24‐hdietary recall, a method prone to misreporting^51^ and possibly not representative of an individual's typical diet. Additionally, specific classes (e.g. soluble vs. insoluble fibre) or sources (e.g. vegetable vs. wholegrain) of dietary fibre may be more efficacious than others for particular applications,^46^ but the method of dietary assessment employed did not differentiate between these. Lastly, inherent to all cross‐sectional data analysis, a causal relationship between dependent and independent outcomes cannot be established.

In summary, higher dietary fibre intakes are associated with a lower body mass and an improvement in body composition (characterized by a higher ratio of lean body mass to fat mass) in adults aged 40 years and older. Moreover, the improvements in body composition with higher dietary fibre intakes are allied with improvements in glucose homeostasis and skeletal muscle strength. Future research should look to evaluate the therapeutic potential of increasing dietary fibre intake (via diet modification and/or supplementation) on skeletal muscle and associated outcomes, with a focus on the preservation of skeletal muscle mass in adults aged 40 years and older.

## Funding

No sources of financial assistance were used to conduct this study or to assist in the preparation of the manuscript.

## Conflicts of interest

J.F., Kevin Murphy, G.F. and E.C. declare that they have no conflicts of interest.

## Author contributions

J.F. and E.S.C. conceived and designed the study. J.F. analysed the data, and K.G.M, G.F. and E.S.C. provided statistical advice. J.F., E.S.C, K.G.M. and G.F. interpreted the results of the analysis. J.F. wrote the initial draft of the manuscript, which was critically revised by E.S.C, K.G.M. and G.F.

## Supporting information


**Table S1:** Variables included in the multiple imputation model.
**Table S2:** Simple and multiple linear regression analyses of dietary fibre intake quarter and body mass components dataset outcomes.
**Table S3:** Simple and multiple linear regression analyses of dietary fibre intake quarters and glucose homeostasis dataset outcomes.
**Table S4:** Simple and multiple linear regression analyses of dietary fibre intake quarters and skeletal muscle strength dataset outcomes.
**Table S5:** Simple and multiple linear regression analyses of dietary fibre intake guideline adherence and all outcomes.
**Figure S1:** Study inclusion flow chart.

## References

[1] Reynolds A , Mann J , Cummings J , Winter N , Mete E , Te Morenga L . Carbohydrate quality and human health: a series of systematic reviews and meta‐analyses. Lancet 2019;393:434–445. 30638909 10.1016/S0140-6736(18)31809-9

[2] Wanders AJ , van den Borne JJGC , de Graaf C , Hulshof T , Jonathan MC , Kristensen M , et al. Effects of dietary fibre on subjective appetite, energy intake and body weight: a systematic review of randomized controlled trials. Obes Rev 2011;12:724–739. 21676152 10.1111/j.1467-789X.2011.00895.x

[3] Bouche C , Rizkalla SW , Luo J , Vidal H , Veronese A , Pacher N , et al. Five‐week, low‐glycemic index diet decreases total fat mass and improves plasma lipid profile in moderately overweight nondiabetic men. Diabetes Care 2002;25:822–828. 11978675 10.2337/diacare.25.5.822

[4] Pal S , Ho S , Gahler RJ , Wood S . Effect on body weight and composition in overweight/obese Australian adults over 12 months consumption of two different types of fibre supplementation in a randomized trial. Nutr Metab Nutr Metab 2016;13:82. 10.1186/s12986-016-0141-7 PMC5114742 27891167

[5] Buckinx F , Landi F , Cesari M , Fielding RA , Visser M , Engelke K , et al. Pitfalls in the measurement of muscle mass: a need for a reference standard. J Cachexia Sarcopenia Muscle 2018;9:269–278. 29349935 10.1002/jcsm.12268 PMC5879987

[6] Baskin KK , Winders BR , Olson EN . Muscle as a “mediator” of systemic metabolism. Cell Metab 2015;21:237–248. 25651178 10.1016/j.cmet.2014.12.021 PMC4398026

[7] Argilés JM , Campos N , Lopez‐Pedrosa JM , Rueda R , Rodriguez‐Mañas L . Skeletal muscle regulates metabolism via interorgan crosstalk: roles in health and disease. J Am Med Dir Assoc 2016;17:789–796. 27324808 10.1016/j.jamda.2016.04.019

[8] DeFronzo RA , Jacot E , Jequier E , Maeder E , Wahren J , Felber JP . The effect of insulin on the disposal of intravenous glucose: results from indirect calorimetry and hepatic and femoral venous catheterization. Diabetes 1981;30:1000–1007. 7030826 10.2337/diab.30.12.1000

[9] DeFronzo RA , Tripathy D . Skeletal muscle insulin resistance is the primary defect in type 2 diabetes. Diabetes Care 2009;32:S157–S163. 19875544 10.2337/dc09-S302 PMC2811436

[10] Deschenes MR . Effects of aging on muscle fibre type and size. Sport Med 2004;34:809–824. 10.2165/00007256-200434120-00002 15462613

[11] Keller K , Engelhardt M . Strength and muscle mass loss with aging process. Age and strength loss. Muscles Ligaments Tendons J 2013;3:346–350. 24596700 PMC3940510

[12] Mitchell WK , Williams J , Atherton P , Larvin M , Lund J , Narici M . Sarcopenia, dynapenia, and the impact of advancing age on human skeletal muscle size and strength; a quantitative review. Front Physiol 2012;3:1–18. 22934016 10.3389/fphys.2012.00260 PMC3429036

[13] US Department of Agriculture, Agricultural Research Service . Nutrient intakes from food and beverages: mean amounts consumed per individual, by gender and age. What We Eat in America, NHANES 2017–2018. 2020. Available from: https://www.ars.usda.gov/

[14] U.S. Department of Agriculture and U.S. Department of Health and Human Services . Dietary guidelines for Americans 2020‐2025. 2020. Available from: https://dietaryguidelines.gov

[15] von Elm E , Altman DG , Egger M , Pocock SJ , Gøtzsche PC , Vandenbroucke JP . The strengthening the reporting of observational studies in epidemiology (STROBE) statement: guidelines for reporting observational studies. Lancet 2007;370:1453–1457. 18064739 10.1016/S0140-6736(07)61602-X

[16] Centers for Disease Control and Prevention . National health and nutrition examination survey. Available from: https://www.cdc.gov/nchs/nhanes/index.htm

[17] Raper N , Perloff B , Ingwersen L , Steinfeldt L , Anand J . An overview of USDA's dietary intake data system. J Food Compos Anal 2004;17:545–555.

[18] U.S. Department of Agriculture ARS . USDA food and nutrient database for dietary studies. Available from: http://www.ars.usda.gov/nea/bhnrc/fsrg

[19] Conway JM , Ingwersen LA , Moshfegh AJ . Accuracy of dietary recall using the USDA five‐step multiple‐pass method in men: an observational validation study. J Am Diet Assoc 2004;104:595–603. 15054345 10.1016/j.jada.2004.01.007

[20] Blanton CA , Moshfegh AJ , Baer DJ , Kretsch MJ . The USDA automated multiple‐pass method accurately estimates group total energy and nutrient intake. J Nutr 2006;136:2594–2599. 16988132 10.1093/jn/136.10.2594

[21] Kim J , Wang Z , Heymsfield SB , Baumgartner RN , Gallagher D . Total‐body skeletal muscle mass: estimation by a new dual‐energy X‐ray absorptiometry method. Am J Clin Nutr 2002;76:378–383. 12145010 10.1093/ajcn/76.2.378

[22] Reginster J , Beaudart C , Buckinx F , Bruyère O . Osteoporosis and sarcopenia. Curr Opin Clin Nutr Metab Care 2016;19:31–36. 26418824 10.1097/MCO.0000000000000230 PMC4888925

[23] University of Oxford Diabetes Trial Unit . HOMA2 calculator. Available from: https://www.dtu.ox.ac.uk/homacalculator/

[24] Norman K , Stobäus N , Gonzalez MC , Schulzke J‐D , Pirlich M . Hand grip strength: outcome predictor and marker of nutritional status. Clin Nutr Elsevier Ltd 2011;30:135–142. 10.1016/j.clnu.2010.09.010 21035927

[25] Centers for Disease Control and Prevention . NHANES survey methods and analytic guidelines. Available from: https://wwwn.cdc.gov/nchs/nhanes/analyticguidelines.aspx

[26] Harrell FE . Regression modeling strategies, 2nd ed. New York: Springer‐Verlag; 2001.

[27] Montiel‐Rojas D , Nilsson A , Santoro A , Franceschi C , Bazzocchi A , Battista G , et al. Dietary fibre may mitigate sarcopenia risk: findings from the NU‐AGE cohort of older European adults. Nutrients 2020;12:1075. 10.3390/nu12041075 PMC7230363 32295007

[28] Slavin JL . Dietary fiber and body weight. Nutrition 2005;21:411–418. 15797686 10.1016/j.nut.2004.08.018

[29] Abate N , Garg A , Peshock RM , Stray‐Gundersen J , Grundy SM . Relationships of generalized and regional adiposity to insulin sensitivity in men. J Clin Invest 1995;96:88–98. 7615840 10.1172/JCI118083 PMC185176

[30] Robertson MD , Bickerton AS , Dennis AL , Vidal H , Frayn KN . Insulin‐sensitizing effects of dietary resistant starch and effects on skeletal muscle and adipose tissue metabolism. Am J Clin Nutr 2005;82:559–567. 16155268 10.1093/ajcn.82.3.559

[31] Bloom I , Shand C , Cooper C , Robinson S , Baird J . Diet quality and sarcopenia in older adults: a systematic review. Nutrients 2018;10:308. 10.3390/nu10030308 PMC5872726 29510572

[32] Berg JM , Tymoczko JL , Stryer L . Glycogen metabolism. In Berg JM , Tymoczko JL , Stryer L , eds. Biochemistry, 5th ed. New York: W. H. Freeman; 2002. p 831–863.

[33] Li S , Guerin‐Deremaux L , Pochat M , Wils D , Reifer C , Miller LE . NUTRIOSE dietary fiber supplementation improves insulin resistance and determinants of metabolic syndrome in overweight men: a double‐blind, randomized, placebo‐controlled study. Appl Physiol Nutr Metab 2010;35:773–782. 21164548 10.1139/H10-074

[34] Solà R , Bruckert E , Valls R‐M , Narejos S , Luque X , Castro‐Cabezas M , et al. Soluble fibre (*Plantago ovata* husk) reduces plasma low‐density lipoprotein (LDL) cholesterol, triglycerides, insulin, oxidised LDL and systolic blood pressure in hypercholesterolaemic patients: a randomised trial. Atherosclerosis 2010;211:630–637. 20413122 10.1016/j.atherosclerosis.2010.03.010

[35] Aliasgharzadeh A , Dehghan P , Gargari BP , Asghari‐Jafarabadi M . Resistant dextrin, as a prebiotic, improves insulin resistance and inflammation in women with type 2 diabetes: a randomised controlled clinical trial. Br J Nutr 2015;113:321–330. 27028002 10.1017/S0007114514003675

[36] Dehghan P , Gargari BP , Jafar‐Abadi MA , Aliasgharzadeh A . Inulin controls inflammation and metabolic endotoxemia in women with type 2 diabetes mellitus: a randomized‐controlled clinical trial. Int J Food Sci Nutr 2014;65:117–123. 24059649 10.3109/09637486.2013.836738

[37] Wilkes EA , Selby AL , Atherton PJ , Patel R , Rankin D , Smith K , et al. Blunting of insulin inhibition of proteolysis in legs of older subjects may contribute to age‐related sarcopenia. Am J Clin Nutr 2009;90:1343–1350. 19740975 10.3945/ajcn.2009.27543

[38] Bach E , Nielsen RR , Vendelbo MH , Moller AB , Jessen N , Buhl M , et al. Direct effects of TNF‐α on local fuel metabolism and cytokine levels in the placebo‐controlled, bilaterally infused human leg: increased insulin sensitivity, increased net protein breakdown, and increased IL‐6 release. Diabetes 2013;62:4023–4029. 23835341 10.2337/db13-0138 PMC3837036

[39] Tieland M , Trouwborst I , Clark BC . Skeletal muscle performance and ageing. J Cachexia Sarcopenia Muscle 2018;9:3–19. 29151281 10.1002/jcsm.12238 PMC5803609

[40] David LA , Maurice CF , Carmody RN , Gootenberg DB , Button JE , Wolfe BE , et al. Diet rapidly and reproducibly alters the human gut microbiome. Nature 2014;505:559–563. 24336217 10.1038/nature12820 PMC3957428

[41] So D , Whelan K , Rossi M , Morrison M , Holtmann G , Kelly JT , et al. Dietary fiber intervention on gut microbiota composition in healthy adults: a systematic review and meta‐analysis. Am J Clin Nutr 2018;107:965–983. 29757343 10.1093/ajcn/nqy041

[42] Frampton J , Murphy KG , Frost G , Chambers ES . Short‐chain fatty acids as potential regulators of skeletal muscle metabolism and function. Nat Metab Springer US 2020;2:840–848. 10.1038/s42255-020-0188-7 32694821

[43] Canfora EE , Jocken JW , Blaak EE . Short‐chain fatty acids in control of body weight and insulin sensitivity. Nat Rev Endocrinol 2015;11:577–591. 26260141 10.1038/nrendo.2015.128

[44] Chen L , Nelson DR , Zhao Y , Cui Z , Johnston JA . Relationship between muscle mass and muscle strength, and the impact of comorbidities: a population‐based, cross‐sectional study of older adults in the United States. BMC Geriatr 2013;13:74. 23865675 10.1186/1471-2318-13-74 PMC3765109

[45] Buigues C , Fernández‐Garrido J , Pruimboom L , Hoogland A , Navarro‐Martínez R , Martínez‐Martínez M , et al. Effect of a prebiotic formulation on frailty syndrome: a randomized, double‐blind clinical trial. Int J Mol Sci 2016;17:932. 10.3390/ijms17060932 PMC4926465 27314331

[46] Stephen AM , Champ MMJ , Cloran SJ , Fleith M , van Lieshout L , Mejborn H , et al. Dietary fibre in Europe: current state of knowledge on definitions, sources, recommendations, intakes and relationships to health. Nutr Res Rev 2017;30:149–190. 28676135 10.1017/S095442241700004X

[47] Woodmansey EJ , McMurdo MET , Macfarlane GT , Macfarlane S . Comparison of compositions and metabolic activities of fecal microbiotas in young adults and in antibiotic‐treated and non‐antibiotic‐treated elderly subjects. Appl Environ Microbiol 2004;70:6113–6122. 15466557 10.1128/AEM.70.10.6113-6122.2004 PMC522128

[48] Salazar N , Arboleya S , Fernández‐Navarro T , de los Reyes‐Gavilán CG , Gonzalez S , Gueimonde M . Age‐associated changes in gut microbiota and dietary components related with the immune system in adulthood and old age: a cross‐sectional study. Nutrients 2019;11:1765. 10.3390/nu11081765 PMC6722604 31370376

[49] Claesson MJ , Jeffery IB , Conde S , Power SE , O'connor EM , Cusack S , et al. Gut microbiota composition correlates with diet and health in the elderly. Nature 2012;488:178–184. 22797518 10.1038/nature11319

[50] Alfa MJ , Strang D , Tappia PS , Graham M , Van Domselaar G , Forbes JD , et al. A randomized trial to determine the impact of a digestion resistant starch composition on the gut microbiome in older and mid‐age adults. Clin Nutr 2018;37:797–807. 28410921 10.1016/j.clnu.2017.03.025

[51] Poslusna K , Ruprich J , de Vries JHM , Jakubikova M , van't Veer P . Misreporting of energy and micronutrient intake estimated by food records and 24 hour recalls, control and adjustment methods in practice. Br J Nutr 2009;101:S73–S85. 19594967 10.1017/S0007114509990602

[52] von Haehling S , Morley JE , Coats AJS , Anker SD . Ethical guidelines for publishing in the Journal of Cachexia, Sarcopenia and Muscle: update 2019. J Cachexia Sarcopenia Muscle 2019;10:1143–1145. 31661195 10.1002/jcsm.12501 PMC6818444

