# Supplementary material for: Higher dietary fibre intake is associated with increased skeletal muscle mass and strength in adults aged 40 years and older
Source: J Cachexia Sarcopenia Muscle. 2021 Sep 29;12(6):2134–44. doi: 10.1002/jcsm.12820 (PMC8718023; doi:10.1002/jcsm.12820)
Supplement: Supplementary file 1 — Table S1: Variables included in the multiple imputation model. Table S2: Simple and multiple linear regression analyses of dietary fibre intake quarter and body mass components dataset outcomes. Table S3: Simple and multiple linear regression analyses of dietary fibre intake quarters and glucose homeostasis dataset outcomes. Table S4: Simple and multiple linear regression analyses of dietary fibre intake quarters and skeletal muscle strength dataset outcomes. Table S5: Simple and multiple linear regression analyses of dietary fibre intake guideline adherence and all outcomes. Figure S1: Study inclusion flow chart. [file JCSM-12-2134-s001.docx]

**Title**

Higher dietary fibre intake is associated with increased skeletal muscle mass and strength in adults aged 40 years and older

**Authors**

James Frampton^1, 2*^, Kevin G. Murphy^2^, Gary Frost^1^, Edward S. Chambers^1^

^1^Section for Nutrition Research, Department of Metabolism, Digestion and Reproduction, Faculty of Medicine, Imperial College London, London W12 0NN, United Kingdom.

^2^Section of Endocrinology and Investigative Medicine, Department of Metabolism, Digestion and Reproduction, Faculty of Medicine, Imperial College London, London W12 0NN, United Kingdom

***Corresponding Author**

James Frampton

Department of Metabolism, Digestion and Reproduction,

Faculty of Medicine,

Imperial College London,

London W12 0NN

United Kingdom

Email: j.frampton17@imperial.ac.uk

**Supplementary Appendix 1:** Multiple imputation procedure

Multiple imputation procedures were based on previous work by Schenker *et al.* [1]. Predictor variables included demographic and socioeconomic variables, body measurements, nutrient intakes, blood tests, health indicators, survey release cycle, sampling weights, strata, and primary sampling units (Table S1).

Prior to imputation, four sex (male, female) by age (40-49, 50-59 years) groups were created. The imputation procedure was then performed separately within each sex-by-age group due to concerns that the distribution of variables included in the model, their availability, and reasons for missingness vary by sex and age.

Multiple imputation with chained equations was used to impute missing values. Normally distributed continuous variables were modelled using linear regression, non-normally distributed continuous and ordinal variables were modelled using predictive mean matching (sampling from a pool of 10 donors [2]), and binary variables were modelled using logistic regression. Data were assumed to be missing at random.

Based on the recommendations of White et al. [3] that the number of imputations should be at least equal to the proportion of incomplete cases (average percentage of missing cases in imputed variables = 21%), 25 imputed datasets were created. Data were only imputed for non-pregnant participants aged ≥40 and ≤59 years that were eligible for dual-energy X-ray absorptiometry measurements and undertook the 24-hour dietary recall interviews (day 1). Multiply imputed values for covariates were included in subsequent analyses. All estimates from imputed datasets were pooled using Rubin’s combination rules [4]. The coefficient of determination (R2) was calculated using the Fisher Z-transformation as recommended by Harel [5].

**Table S1:** Variables included in the multiple imputation model

| **Variable (units)** | **NHANES variable label^a^** | **% missing** | **Model** |
| --- | --- | --- | --- |
| age (years) | ridageyr | 0.00 | - |
| annual household income ($) | indhhin2 | 6.38 | PMM |
| arm circumference (cm) | bmxarmc | 3.28 | regression |
| body mass index (kg/m^2^) | bmxbmi | 1.05 | PMM |
| body mass (kg) | bmxwt | 0.92 | PMM |
| bone mineral content (g) | dxdtobmc | 22.43 | regression |
| bone mineral density (g/cm^2^) | dxdtobmd | 22.43 | regression |
| calcium supplements (mg) | dsqtcalc | 63.01 | PMM |
| daily alcohol (g) | dr1talco | 7.22 | PMM |
| daily carbohydrate intake (g) | dr1tcarb | 7.19 | PMM |
| daily fat intake (g) | dr1ttfat | 7.19 | PMM |
| daily fibre intake (g) | dr1tfibe | 7.19 | PMM |
| daily protein intake (g) | dr1tprot | 7.19 | PMM |
| daily total energy intake (kcal) | dr1tkcal | 7.19 | PMM |
| data release cycle | sddsrvyr | 0.00 | - |
| diastolic blood pressure (mmHg) | N/A^b^ | 3.51 | PMM |
| education level | dmdeduc2 | 0.03 | PMM |
| ethnicity | ridreth1 | 0.00 | - |
| fasting glucose (mmol/L) | lbdglusi | 53.51 | PMM |
| fasting insulin (pmol/L) | lbdinsi | 54.72 | PMM |
| full sample 8 year dietary day 1 sample weight | N/A^c^ | 0.00 | - |
| full sample 8 year MEC exam weight | N/A^c^ | 0.00 | - |
| general health condition | hsd010 | 9.26 | PMM |
| height (cm) | bmxht | 0.91 | regression |
| HOMA2-B (AU) | N/A^d^ | 54.80 | PMM |
| HOMA2-IR (AU) | N/A^d^ | 54.77 | PMM |
| HOMA2-S (AU) | N/A^d^ | 54.80 | PMM |
| LDL-cholesterol (mmol/L) | lbdldlsi | 56.07 | PMM |
| left arm lean excl BMC (g) | dxdlale | 15.19 | regression |
| left leg lean excl BMC (g) | dxdllle | 16.71 | regression |
| past 30 day milk product consumption | dbq197 | 0.00 | - |
| primary sampling units | sdmvpsu | 0.00 | - |
| ratio of family income to poverty | indfmpir | 9.49 | regression |
| right arm lean excl BMC (g) | dxdrale | 15.92 | regression |
| right leg lean excl BMC (g) | dxdrlle | 17.08 | regression |
| sampling strata | sdmvstra | 0.00 | - |
| sedentary activity (mins) | pad680 | 0.60 | regression |
| smoking status | N/A^e^ | 0.01 | logistic regression |
| systolic blood pressure (mmHg) | N/A^f^ | 3.51 | PMM |
| testosterone (ng/dL) | lbxtst | 28.31 | PMM |
| total cholesterol (mmol/L) | lbdtcsi | 5.23 | PMM |
| total fat (g) | dxdtofat | 22.65 | PMM |
| total lean mass excluding BMC (g) | dxdtole | 21.21 | regression |
| triglyceride (mmol/L) | lbdtrsi | 55.15 | PMM |
| trunk fat (g) | dxxtrfat | 18.10 | PMM |
| vitamin D (D2 + D3) supplements (mcg) | dsqtvd | 64.45 | PMM |
| waist circumference (cm) | bmxwaist | 4.20 | regression |
|  |  |  |  |
| **Average % missing:** |  | **20.59** |  |

^a^refers to the label used in the NHANES database (https://wwwn.cdc.gov/Nchs/Nhanes/search/default.aspx). ^b^calculated as an average of bpxdi1, bpxdi2, bpxdi3, and bpxdi4. ^C^calculated using 2-year weights (see main text). ^d^calculated using the HOMA2 calculator (see main text). ^e^created using smq020 and smq040 (https://wwwn.cdc.gov/nchs/nhanes/tutorials/Module1.aspx; see ‘Skip Patterns in NHANES Data’). f ^b^calculated as an average of bpxsy1, bpxsy2, bpxsy3, and bpxsy4 as % missing was calculated from the number of missing values for participants that were eligible for dual-energy X-ray absorptiometry measurements and 24-hour dietary recall interviews (day 1), and aged between 40 and 59 years old (inclusive). Average % missing was calculated from variables that were imputed (i.e., % missing > 0). AU, arbitrary units; BMC, bone mineral content; HOMA2-B, updated homeostasis model assessment - beta cell function; HOMA2-IR, updated homeostasis model assessment - insulin resistance; HOMA2-S, updated homeostasis model assessment - insulin sensitivity; LDL, low-density lipoprotein; MEC, mobile examination center; N/A, not available; NHANES, national health and nutrition examination survey; PMM, predictive mean matching.

**Supplementary Appendix 2:** Study inclusion flow chart.


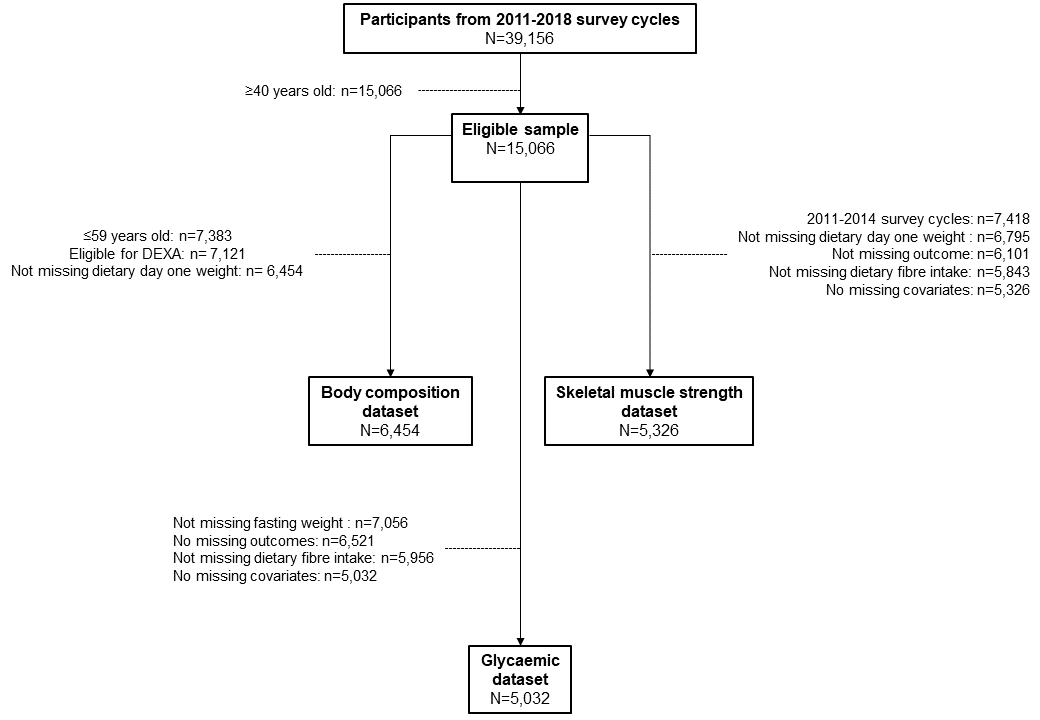


**Figure S1:** Study inclusion flow chart

**Supplementary Appendix 3:** Results of Model 4 for body mass components and skeletal muscle strength datasets

| **Outcomes** | **Model 3^a^** | | |  | **Model 4^b^** | | |
| --- | --- | --- | --- | --- | --- | --- | --- |
|  | **β (95% CI)** | **P value** | **R^2^** |  | **β (95% CI)** | **P value** | **R^2^** |
| Body mass  (kg) | -0.20 (-0.28, -0.11) | <0.001 | 0.18 |  | -0.15 (-0.23, -0.06) | 0.001 | 0.31 |
| BMI  (kg/m^2^) | -0.08 (-0.10, -0.05) | <0.001 | 0.09 |  | -0.06 (-0.09, -0.03) | <0.001 | 0.37 |
| Relative total lean mass  (g/kg BM) | 0.69 (0.48, 0.89) | <0.001 | 0.57 |  | 0.61 (0.40, 0.82) | <0.001 | 0.60 |
| Relative appendicular lean mass  (g/kg BM) | 0.34 (0.23, 0.45) | <0.001 | 0.63 |  | 0.29 (0.18, 0.41) | <0.001 | 0.65 |
| Relative bone mineral content  (g/kg BM) | 0.05 (0.02, 0.07) | <0.001 | 0.11 |  | 0.03 (0.01, 0.06) | 0.009 | 0.19 |
| Relative total fat  (g/kg BM) | -0.68 (-0.89, -0.47) | <0.001 | 0.56 |  | -0.60 (-0.81, -0.39) | <0.001 | 0.59 |
| Relative trunk fat  (g/kg BM) | -0.48 (-0.63, -0.33) | <0.001 | 0.29 |  | -0.40 (-0.55, -0.25) | <0.001 | 0.37 |
|  |  |  |  |  |  |  |  |
| Relative combined grip strength  (kg/kg BM) | 0.002 (0.001, 0.003) | <0.001 | 0.40 |  | 0.001 (0.000, 0.003) | 0.037 | 0.48 |

^a^Adjusted for gender, age, ethnicity, socioeconomic status, smoking status, sedentary activity, total energy intake, total alcohol intake, percent energy from protein, percent energy from carbohydrate, and percent energy from fat. ^b^Adjusted for gender, age, ethnicity, socioeconomic status, smoking status, sedentary activity, total energy intake, total alcohol intake, percent energy from protein, percent energy from carbohydrate, percent energy from fat, and HOMA2-IR. BMI, body mass index; HOMA2-IR, updated homeostasis model assessment - insulin resistance. Results from Model 3 provided for comparison.

**Supplementary Appendix 4:** Dietary fibre intake quarter analysis

Dietary fibre quarters using the 25th, 50th and 75th percentiles were generated from dietary fibre intake as estimated by the 24-hour dietary recall (day 1).

Simple and multiple linear regression analyses were used to examine the association between dietary fibre intake quarter and outcome variables. Model 1 was an unadjusted model. Model 2 was adjusted for gender, age, ethnicity, socioeconomic status, smoking status, and sedentary activity. Model 3 was adjusted for gender, age, ethnicity, socioeconomic status, smoking status, sedentary activity, total energy intake, total alcohol intake, percent energy from protein, percent energy from carbohydrate, and percent energy from fat. Orthogonal polynomial contrasts were used to perform linear trend analyses across dietary fibre quarters. Results from analyses are presented by dataset in Tables S2-S4.

**Table S2:** Simple and multiple linear regression analyses of dietary fibre intake quarter and body mass components dataset outcomes.

| **Outcome** | **Q1**  (0-9.6 g/day) | | **Q2**  (9.7-15.2 g/day) | | **Q3**  (15.3-22.5 g/day) | | **Q4**  (22.6-107.0 g/day) | | **R^2^** | **P for trend^a^** |
| --- | --- | --- | --- | --- | --- | --- | --- | --- | --- | --- |
|  | **β (95% CI)** | **P value** | **β (95% CI)** | **P value** | **β (95% CI)** | **P value** | **β (95% CI)** | **P value** |  |  |
| Model 1^b^ |  |  |  |  |  |  |  |  |  |  |
| Body mass  (kg) | Reference | | 2.62 (0.52, 4.71) | 0.015 | 1.67 (-0.70, 4.03) | 0.165 | 2.33 (0.43, 4.23) | 0.017 | <0.01 | 0.043 |
| BMI  (kg/m^2^) | Reference | | 0.52 (-0.21, 1.25) | 0.161 | -0.20 (-0.99, 0.58) | 0.607 | -0.69 (-1.39, 0.01) | 0.052 | <0.01 | 0.013 |
| Relative total lean mass  (g/kg BM) | Reference | | 5.63 (-2.10, 13.36) | 0.150 | 22.64 (14.14, 31.15) | <0.001 | 38.66 (30.43, 46.88) | <0.001 | 0.04 | <0.001 |
| Relative appendicular lean mass  (g/kg BM) | Reference | | 6.24 (1.93, 10.56) | 0.005 | 14.32 (9.43, 19.21) | <0.001 | 23.65 (19.26, 28.04) | <0.001 | 0.04 | <0.001 |
| Relative bone mineral content  (g/kg BM) | Reference | | -0.27 (-0.93, 0.40) | 0.421 | 0.40 (-0.30, 1.09) | 0.255 | 0.82 (0.18, 1.46) | <0.001 | <0.01 | 0.003 |
| Relative total fat  (g/kg BM) | Reference | | -5.61 (-13.45, 2.22) | 0.157 | -22.75 (-31.51, -14.00) | <0.001 | -38.67 (-47.27, -30.06) | <0.001 | 0.03 | <0.001 |
| Relative trunk fat  (g/kg BM) | Reference | | -3.67 (-8.36, 1.01) | 0.122 | -11.12 (-16.12, -6.11) | <0.001 | -18.79 (-23.18, -14.40) | <0.001 | 0.03 | <0.001 |
| Model 2^c^ |  |  |  |  |  |  |  |  |  |  |
| Body mass  (kg) | Reference | | 1.20 (-0.67, 3.07) | 0.205 | -0.30 (-2.51, 1.91) | 0.788 | -0.42 (-2.15, 1.30) | 0.626 | 0.16 | 0.328 |
| BMI  (kg/m^2^) | Reference | | 0.36 (-0.34, 1.07) | 0.304 | -0.25 (-1.01, 0.52) | 0.524 | -0.65 (-1.27, -0.02) | 0.042 | 0.07 | 0.015 |
| Relative total lean mass  (g/kg BM) | Reference | | 0.88 (-5.03, 6.79) | 0.766 | 9.76 (3.34, 16.18) | 0.004 | 15.20 (10.48, 19.93) | <0.001 | 0.56 | <0.001 |
| Relative appendicular lean mass  (g/kg BM) | Reference | | 2.49 (-0.31, 5.30) | 0.080 | 6.31 (2.89, 9.72) | <0.001 | 9.59 (7.18, 12.01) | <0.001 | 0.62 | <0.001 |
| Relative bone mineral content  (g/kg BM) | Reference | | -0.33 (-0.99, 0.33) | 0.325 | 0.22 (-0.49, 0.93) | 0.536 | 0.46 (-0.15, 1.07) | 0.139 | 0.10 | 0.060 |
| Relative total fat  (g/kg BM) | Reference | | -0.93 (-7.01, 5.16) | 0.761 | -9.94 (-16.65, -3.22) | 0.004 | -15.21 (-20.19, -10.23) | <0.001 | 0.55 | <0.001 |
| Relative trunk fat  (g/kg BM) | Reference | | -2.50 (6.57, 1.56) | 0.223 | -7.90 (-12.43, -3.37) | 0.001 | -12.39 (-16.00, -8.78) | <0.001 | 0.10 | <0.001 |
|  |  |  |  |  |  |  |  |  |  |  |
| Model 3^d^ |  |  |  |  |  |  |  |  |  |  |
| Body mass  (kg) | Reference | | -0.46 (-2.14, 1.22) | 0.584 | -2.97 (-5.25, -0.68) | 0.012 | -4.41 (-6.53, -2.28) | <0.001 | 0.18 | <0.001 |
| BMI  (kg/m^2^) | Reference | | -0.15 (-0.79, 0.49) | 0.634 | -1.07 (-1.84, -0.30) | 0.007 | -1.88 (-2.62, -1.15) | <0.001 | 0.09 | <0.001 |
| Relative total lean mass  (g/kg BM) | Reference | | 1.83 (-3.65, 7.30) | 0.507 | 11.59 (4.99, 18.18) | 0.001 | 17.56 (11.56, 23.55) | <0.001 | 0.57 | <0.001 |
| Relative appendicular lean mass  (g/kg BM) | Reference | | 2.67 (0.02, 5.32) | 0.048 | 6.75 (3.30, 10.20) | <0.001 | 10.02 (6.84, 13.21) | <0.001 | 0.63 | <0.001 |
| Relative bone mineral content  (g/kg BM) | Reference | | -0.13 (-0.78, 0.53) | 0.700 | 0.56 (-0.22, 1.34) | 0.158 | 0.96 (0.21, 1.70) | 0.012 | 0.11 | 0.006 |
| Relative total fat  (g/kg BM) | Reference | | -1.81 (-7.47, 3.84) | 0.524 | -11.65 (18.57, -4.74) | 0.001 | -17.36 (-23.51, -11.23) | <0.001 | 0.56 | <0.001 |
| Relative trunk fat  (g/kg BM) | Reference | | -3.02 (-6.92, 0.87) | 0.126 | -8.92 (-13.70, -4.14) | <0.001 | -13.61 (-18.03, -9.20) | <0.001 | 0.29 | <0.001 |

^a^P value for linear trend across quarters using orthogonal polynomial contrasts.^b^Unadjusted model. ^c^Adjusted for gender, age, ethnicity, socioeconomic status, smoking status, and sedentary activity. ^d^Adjusted for gender, age, ethnicity, socioeconomic status, smoking status, sedentary activity, total energy intake, total alcohol intake, percent energy from protein, percent energy from carbohydrate, and percent energy from fat. BMI, body mass index. Q1, quarter 1; Q2, quarter 2; Q3, quarter 3; Q4, quarter 4. Dietary fibre intake quarter boundaries (g/day) are presented below corresponding quarters in parentheses.

**Table S3:** Simple and multiple linear regression analyses of dietary fibre intake quarters and glucose homeostasis dataset outcomes.

| **Outcome** | **Q1**  (0-9.6 g/day) | | **Q2**  (9.7-14.9 g/day) | | **Q3**  (15.0-22.1 g/day) | | **Q4**  (22.2-107.0 g/day) | | **R^2^** | **P for trend^a^** |
| --- | --- | --- | --- | --- | --- | --- | --- | --- | --- | --- |
|  | **β (95% CI)** | **P value** | **β (95% CI)** | **P value** | **β (95% CI)** | **P value** | **β (95% CI)** | **P value** |  |  |
| Model 1^b^ |  |  |  |  |  |  |  |  |  |  |
| Fasting glucose  (mmol/L) | Reference | | 0.03 (-0.14, 0.21) | 0.700 | 0.02 (-0.18, 0.21) | 0.855 | 0.07 (-0.13, 0.26) | 0.489 | <0.01 | 0.527 |
| Fasting insulin  (pmol/L) | Reference | | -1.72 (-9.83, 6.40) | 0.674 | -0.53 (-8.13, 7.07) | 0.890 | 2.40 (-4.63, 9.43) | 0.498 | <0.01 | 0.492 |
| HOMA2-IR  (AU) | Reference | | -0.05 (-0.24, 0.13) | 0.577 | -0.04 (-0.22, 0.13) | 0.641 | 0.05 (-0.11, 0.22) | 0.540 | <0.01 | 0.558 |
| Model 2^c^ |  |  |  |  |  |  |  |  |  |  |
| Fasting glucose  (mmol/L) | Reference | | 0.05 (-0.12, 0.21) | 0.569 | -0.00 (-0.20, 0.19) | 0.980 | -0.01 (-0.21, 0.18) | 0.894 | 0.03 | 0.769 |
| Fasting insulin  (pmol/L) | Reference | | -1.06 (-8.89, 6.77) | 0.788 | -1.77 (-9.68, 6.14) | 0.656 | -0.84 (-7.90, 6.22) | 0.813 | 0.03 | 0.798 |
| HOMA2-IR  (AU) | Reference | | -0.03 (-0.2, 0.14) | 0.696 | -0.07 (-0.25, 0.11) | 0.446 | -0.02 (-0.19, 0.14) | 0.784 | 0.03 | 0.722 |
| Model 3^d^ |  |  |  |  |  |  |  |  |  |  |
| Fasting glucose  (mmol/L) | Reference | | 0.02 (-0.15 0.18) | 0.856 | -0.05 (-0.23, 0.14) | 0.634 | -0.08 (-0.32, 0.15) | 0.479 | 0.04 | 0.389 |
| Fasting insulin  (pmol/L) | Reference | | -6.50 (-14.50, 1.50) | 0.110 | -10.65 (-18.11, -3.81) | 0.006 | -14.77 (-22.46, -7.08) | <0.001 | 0.05 | <0.001 |
| HOMA2-IR  (AU) | Reference | | -0.16 (-0.34, 0.02) | 0.079 | -0.27 (-0.44, -0.11) | 0.001 | -0.35 (-0.53, -0.16) | <0.001 | 0.04 | <0.001 |

^a^P value for linear trend across quarters using orthogonal polynomial contrasts.^b^Unadjusted model. ^c^Adjusted for gender, age, ethnicity, socioeconomic status, smoking status, and sedentary activity. ^d^Adjusted for gender, age, ethnicity, socioeconomic status, smoking status, sedentary activity, total energy intake, total alcohol intake, percent energy from protein, percent energy from carbohydrate, and percent energy from fat. AU, arbitrary units; HOMA2-IR, updated homeostasis model assessment - insulin resistance. Q1, quarter 1; Q2, quarter 2; Q3, quarter 3; Q4, quarter 4. Dietary fibre intake quarter boundaries (g/day) are presented below corresponding quarter in parentheses.

**Table S4:** Simple and multiple linear regression analyses of dietary fibre intake quarters and skeletal muscle strength dataset outcomes.

| **Outcome** | **Q1**  (0-9.8 g/day) | | **Q2**  (9.9-15.0 g/day) | | **Q3**  (15.1-22.2 g/day) | | **Q4**  (22.3-107.0 g/day) | | **R^2^** | **P for trend^a^** |
| --- | --- | --- | --- | --- | --- | --- | --- | --- | --- | --- |
|  | **β (95% CI)** | **P value** | **β (95% CI)** | **P value** | **β (95% CI)** | **P value** | **β (95% CI)** | **P value** |  |  |
| Model 1^b^ |  |  |  |  |  |  |  |  |  |  |
| Relative combined grip strength  (kg/kg BM) | Reference | | -0.006 (-0.044, 0.0316) | 0.750 | 0.0406 (0.016, 0.066) | 0.002 | 0.095 (0.066, 0.132) | <0.001 | 0.03 | <0.001 |
| Model 2^c^ |  |  |  |  |  |  |  |  |  |  |
| Relative combined grip strength  (kg/kg BM) | Reference | | -0.007 (-0.036, 0.021) | 0.599 | 0.012 (-0.006, 0.030) | 0.189 | 0.037 (0.016, 0.057) | 0.001 | 0.39 | <0.001 |
| Model 3^d^ |  |  |  |  |  |  |  |  |  |  |
| Relative combined grip strength  (kg/kg BM) | Reference | | -0.001 (0.028, 0.026) | 0.944 | 0.021 (-0.000, 0.042) | 0.054 | 0.048 (0.025, 0.072) | <0.001 | 0.40 | <0.001 |

^a^P value for linear trend across quarters using orthogonal polynomial contrasts.^b^Unadjusted model. ^c^Adjusted for gender, age, ethnicity, socioeconomic status, smoking status, and sedentary activity. ^d^Adjusted for gender, age, ethnicity, socioeconomic status, smoking status, sedentary activity, total energy intake, total alcohol intake, percent energy from protein, percent energy from carbohydrate, and percent energy from fat. Q1, quarter 1; Q2, quarter 2; Q3, quarter 3; Q4, quarter 4. Dietary fibre intake quarter boundaries (g/day) are presented below corresponding quarter in parentheses.

**Supplementary Appendix 5:** Dietary fibre intake guideline analysis

Participants were divided into two groups: (i) participants meeting the USDA (14g dietary fibre per 1000kcal), and (ii) participants not meeting the USDA guidelines. This grouping was based on dietary fibre intake as estimated by the 24-hour dietary recall (day 1).

Simple and multiple linear regression analyses were used to examine the association between dietary fibre intake guideline adherence and outcome variables. Model 1 was an unadjusted model. Model 2 was adjusted for gender, age, ethnicity, socioeconomic status, smoking status, and sedentary activity. Model 3 was adjusted for gender, age, ethnicity, socioeconomic status, smoking status, sedentary activity, total energy intake, total alcohol intake, percent energy from protein, percent energy from carbohydrate, and percent energy from fat. Results from analyses are presented by dataset in Tables S5.

**Table S5:** Simple and multiple linear regression analyses of dietary fibre intake guideline adherence and all outcomes

| **Outcomes** | **Model 1^a^** | | | **Model 2^b^** | | | | | **Model 3^c^** | | |
| --- | --- | --- | --- | --- | --- | --- | --- | --- | --- | --- | --- |
|  | **β (95% CI)** | **P value** | **R^2^** |  | **β (95% CI)** | **P value** | **R^2^** |  | **β (95% CI)** | **P value** | **R^2^** |
| Body mass  (kg) | -7.13 (-9.42, -4.84) | <0.001 | 0.01 |  | -4.71 (-6.84, -2.59) | <0.001 | 0.16 |  | -3.57 (-5.86, -1.27) | 0.003 | 0.18 |
| BMI  (kg/m^2^) | -1.71 (-2.43, -0.99) | <0.001 | 0.01 |  | -1.75 (-2.48, -1.02) | <0.001 | 0.08 |  | -1.53 (-2.35, -0.72) | <0.001 | 0.09 |
| Relative total lean mass  (g/kg BM) | -3.69 (-12.20, 4.82) | 0.389 | <0.01 |  | 10.01 (4.15, 15.88) | 0.001 | 0.56 |  | 10.99 (4.58, 17.40) | 0.001 | 0.57 |
| Relative appendicular lean mass  (g/kg BM) | -4.10 (-9.24, 1.04) | 0.116 | <0.01 |  | 4.37 (0.83, 7.92) | 0.017 | 0.62 |  | 4.80 (0.99, 8.61) | 0.014 | 0.62 |
| Relative bone mineral content  (g/kg BM) | 0.90 (0.25, 1.54) | 0.007 | <0.01 |  | 1.19 (0.54, 1.84) | 0.001 | 0.10 |  | 1.16 (0.46, 1.87) | 0.002 | 0.11 |
| Relative total fat  (g/kg BM) | 4.03 (-4.70, 12.76) | 0.359 | <0.01 |  | -9.91 (-16.02, -3.81) | 0.002 | 0.55 |  | -10.72 (-17.40, -4.03) | 0.002 | 0.56 |
| Relative trunk fat  (g/kg BM) | -3.41 (-8.10, 1.28) | 0.151 | <0.01 |  | -9.84 (-14.52, -5.17) | <0.001 | 0.28 |  | -9.85 (-15.00, -4.70) | <0.001 | 0.29 |
|  |  |  |  |  |  |  |  |  |  |  |  |
| Fasting glucose  (mmol/L) | -0.20 (-0.36, -0.05) | 0.013 | <0.01 |  | -0.27 (-0.42, -0.11) | 0.001 | 0.03 |  | -0.27 (-0.43, -0.10) | 0.002 | 0.04 |
| Fasting insulin  (pmol/L) | -8.09 (-15.52, -0.65) | 0.033 | <0.01 |  | -10.08 (-18.21, -1.96) | 0.016 | 0.03 |  | -9.88 (-18.12, -1.65) | 0.019 | 0.04 |
| HOMA2-IR  (AU) | -0.21 (-0.37, -0.04) | 0.014 | <0.01 |  | -0.25 (-0.43, -0.07) | 0.007 | 0.03 |  | -0.25 (-0.43, -0.07) | 0.008 | 0.04 |
|  |  |  |  |  |  |  |  |  |  |  |  |
| Relative combined grip strength  (kg/kg BM) | -0.001 (-0.028, 0.025) | 0.922 | <0.01 |  | 0.029 (0.003, 0.055) | 0.028 | 0.39 |  | 0.033 (0.007, 0.058) | 0.013 | 0.40 |

^a^Unadjusted model. ^b^Adjusted for gender, age, ethnicity, socioeconomic status, smoking status, and sedentary activity. ^c^Adjusted for gender, age, ethnicity, socioeconomic status, smoking status, sedentary activity, total energy intake, total alcohol intake, percent energy from protein, percent energy from carbohydrate, and percent energy from fat. AU, arbitrary units; BMI, body mass index; HOMA2-IR, updated homeostasis model assessment - insulin resistance.

**REFERENCES**

1. Schenker N, Borrud LG, Burt VL, Curtin LR, Flegal KM, Hughes J, et al. Multiple imputation of missing dual-energy X-ray absorptiometry data in the National Health and Nutrition Examination Survey. Stat Med. 2011;30:260–76.

2. Morris TP, White IR, Royston P. Tuning multiple imputation by predictive mean matching and local residual draws. BMC Med Res Methodol. 2014;14:75.

3. White IR, Royston P, Wood AM. Multiple imputation using chained equations: Issues and guidance for practice. Stat Med. 2011;30:377–99.

4. Rubin DB. Multiple imputation for nonresponse in surveys. New York: Wiley; 1987.

5. Harel O. The estimation of R2 and adjusted R2 in incomplete data sets using multiple imputation. J Appl Stat. 2009;36:1109–18.
